# 

**Published:** 1886-11

**Authors:** 


COLLABORATORS.

CHICAGO:

PROFESSOR J. A. ALLEN, M. D., LL. D.
PROFESSOR E. ANDREWS, M. D., LL. D.
PROFESSOR JOHN BARTLETT, M. D.
PROFESSOR W. H. BYFORD, A. M., M. D.
PROFESSOR O. C. DEWOLF, A. M., M. D.
PROFESSOR E. C. DUDLEY, A. B., M. D.
PROFESSOR J. H. ETHERIDGE, A. M., M. D.
PROFESSOR C. FENGER, M. D.
PROFESSOR J. N. HYDE, A. M., M. D.
PROFESSOR E. F. INGALS, A. M., M.D.
PROFESSOR W. W. JAGGARD, A. M., M. D.
PROFESSOR H. A. JOHNSON, M. D., LL. D.
PROFESSOR CHARLES GILMAN SMITH, A. M., M.D.

MILWAUKEE, WISCONSIN:
PROFESSOR N. SENN, M. D.

WASHINGTON, D. C.:
S. O. RICHEY, M. D.

UNITED STATES ARMY:
SURGEON, D. L. HUNTINGTON, M. D.

UNITED STATES NAVY:
MEDICAL DIRECTOR, J. M. BROWNE, M. D.
MEDICAL DIRECTOR, A. L. GIHON, A. M., M. D.

